# Strain-Engineered Adaptive 2D Photodetectors: A New Approach to Miniaturized Reconstructive Spectrometry

**DOI:** 10.1021/acs.nanolett.5c02470

**Published:** 2025-07-12

**Authors:** Thiago L. Vasconcelos, Simeon N. Vladimirov, Thomas Pucher, Sergio Puebla, Carmen Munuera, Eduardo R. Hernández, Andres Castellanos-Gomez

**Affiliations:** † 2D Foundry Research Group, Instituto de Ciencia de Materiales de Madrid (ICMM-CSIC), Madrid E-28049, Spain; ‡ Materials Metrology Division, Instituto Nacional de Metrologia Qualidade e Tecnologia (INMETRO), Duque de Caxias, Rio de Janeiro 25250-020, Brazil; § Computational Materials Laboratory, Instituto de Ciencia de Materiales de Madrid Madrid (ICMM-CSIC), Madrid E-28049, Spain

**Keywords:** reconstructive spectrometry, straintronics, 2D materials, neural networks, miniaturized devices, microspectrometers

## Abstract

We present adaptive, strain-engineered photodetectors based on 2D semiconductors. Our devices present a footprint of only about 40 × 20 μm^2^, and they are fabricated on transparent polypropylene substrates and integrate lithographically designed microheaters that induce controllable biaxial strain via thermal actuation, thus leading to a red shift in their spectral response. This strain-induced tunability is combined with a machine learning-based spectral reconstruction approach, where a neural network is trained on an extensive data set of synthetic spectra and corresponding photocurrents generated from measured device responsivities under varying strain conditions. Although the system demonstrates spectral reconstruction over a relatively narrow range, it provides a compelling proof-of-concept for a miniaturized adaptive spectrometer.

## Graphical abstract

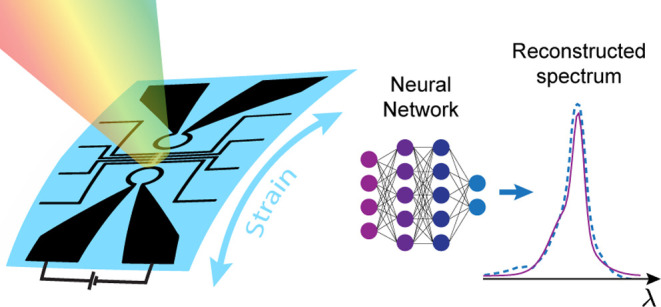

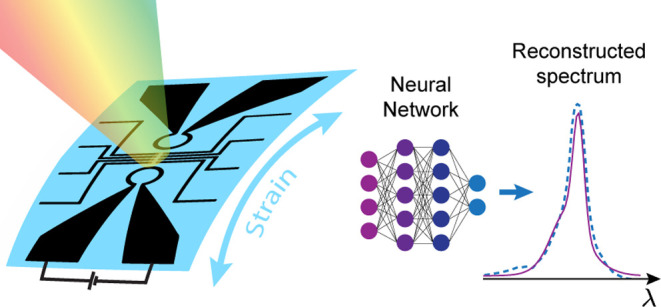

The emerging field of straintronics, i.e., the engineering of electronic properties through the application of mechanical strain, enabling dynamic control over device performance, offers a compelling route to realizing tunable optoelectronic devices.^1–3^ By mechanically deforming a material, its electronic band structure can be modulated, leading to significant changes in both its optical and electrical behavior.^4^ This approach has attracted considerable interest for the development of adaptive photodetectors, where device performance can be continuously tuned via the application of strain.^5,6^ In essence, straintronics leverages the mechanical flexibility of materials to achieve functionalities that are unattainable by conventional static designs.

Two-dimensional (2D) materials are particularly well-suited for strain-engineered applications due to their exceptional resilience to large deformations and the high sensitivity of their electronic band structures to applied strain.^7–9^ Unlike bulk 3D semiconductors, many 2D materials can sustain substantial tensile and compressive strains without mechanical failure,^10,11^ while their bandgaps and optical absorption spectra exhibit pronounced modulations under strain. Such characteristics make 2D semiconductors ideal candidates for adaptive photodetectors in which the active layer simultaneously serves as the sensing element and the tunable medium.

Recent advances have demonstrated that strain-tunable photodetectors can achieve a significant modulation of key device parameters (including responsivity, spectral bandwidth, and response time) through controlled mechanical deformation. For instance, strain-engineered MoTe_2_ photodetectors have shown enhanced responsivity in the near-infrared range,^6^ while monolayer MoS_2_ devices exhibit marked increases in photoresponse under applied strain.^5,12^ Additional studies on In_2_S_3_ and InSe-based devices further underscore the potential of strain engineering to enhance the photocurrent generation and to extend the operational range of photodetectors by dynamically tuning their band structures and optical properties.^13,14^

A particularly attractive application of tunable photodetectors is in the field of spectral sensing. Reconstructive spectrometers, i.e., miniaturized devices that employ computational algorithms to reconstruct spectral information from encoded signals, offer a pathway toward compact and portable spectroscopic systems.^15–20^ By integrating adaptive photodetectors with tunable spectral responses, it becomes possible to exert real-time control over a single sensing pixel, thereby enabling on-demand spectral reconstruction at a minimum footprint.^21–27^ While the state of the art in single-pixel reconstructive spectrometry has mostly focused on fixed or electronically tuned encoding elements, the intrinsic strain tunability of the sensing pixel remains unexplored in this context.

In this work, we introduce a novel approach that integrates strain-engineered adaptive photodetectors with reconstructive spectrometry. Leveraging the high strain tolerance and sensitive band structure modulation of 2D semiconductors, we demonstrate that mechanical actuation, implemented via integrated microheaters, can dynamically tune the spectral response of a photodetector. This tunability is then harnessed to realize a miniaturized reconstructive spectrometer, thereby opening a new avenue for compact, adaptive spectral sensing systems.^15,17^ In contrast to conventional matrix-inversion techniques used in most gate-tunable van der Waals spectrometers, our system employs a neural network-based reconstruction approach, similar to the work of Darweesh et al.^26^ A point of contrast with that work is that we trained our neural network on a database consisting of thousands of synthetic spectra and corresponding photocurrents obtained from them and the measured photocurrent responsivity of the device, rather than a limited number of experimental spectra, as explained in more detail below. This novel training strategy enables the accurate reconstruction of the incident spectrum from the strain-induced photocurrent shifts, highlighting the potential of our platform for next-generation spectroscopic applications.

We fabricated strain-engineered adaptive photodetectors on polypropylene (PP) substrates by using a combination of maskless photolithography and a dry deterministic transfer technique. Prior to microfabrication, the PP substrates were planarized by heating near their melting temperature (145 °C) and applying mechanical pressure using a silicon wafer and weight assembly (see the Supporting Information). We then performed maskless photolithography followed by gold deposition and lift-off to pattern the microheaters and the photodetector drain–source electrodes on top of the PP substrate (Figure 1a,b). High-quality 2D semiconductor flakes (WS_2_ and WSe_2_, obtained from HQ Graphene) were mechanically exfoliated using Nitto SPV224 tape and transferred onto a gel film substrate (WF 4 × 6.0 mil, by GelPak). Flakes with the desired morphology and number of layers were identified using transmission-mode optical microscopy and differential microreflectance spectroscopy.^28,29^

**Figure 1:**
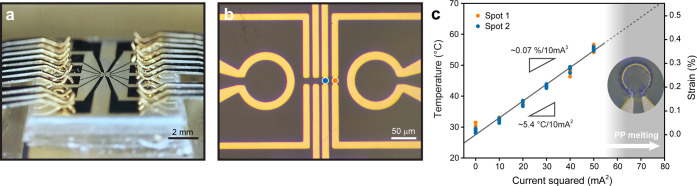
(a) Photograph of a strain-engineered adaptive photodetector device under test. (b) Optical microscopy image of the patterned microheater actuators for local thermal expansion and the four channels, composed of drain–source pairs of electrodes, prior to the 2D material transfer. (c) Calibration curve showing the temperature (and corresponding substrate thermal expansion, assumed to be transferred as biaxial strain to the device) at the MoS_2_ monolayer as a function of the applied microheater current squared (mA^2^). By performing a linear regression fit to the data from spot 2 (blue marker position in panel b), we estimate a temperature variation of approximately 5.4 °C and an induced strain of about 0.07% per 10 mA^2^ applied to the microheater actuator. The inset in panel c shows an optical image of one of the actuators after operation with an applied current exceeding 60 mA^2^, indicating a safe operating limit.

We selected flakes no wider than 20 μm at their thinnest region to be placed between the drain and source electrodes and with a maximum thickness of seven layers to ensure a significant strain-induced energy shift. However, we avoided monolayers as this would make subsequent material transfers more challenging without damaging the previously transferred material. The chosen flakes were then transferred, using deterministic transfer technique, onto prepatterned source–drain electrodes on the substrate.^30^

In a final step the devices were encapsulated using a recently developed Formvar polymer technique to improve stability during thermal actuation, increase the strain transfer by about 2-fold, and enhance the optoelectronic performance of the 2D material (more details in the Supporting Information).^31^

Figure 1 summarizes the device architecture and strain calibration. Panels a and b present optical images of a representative device. In panel a, a home-built probe card with gold-coated copper contacts is pressed onto the terminals of the microfabricated circuit on the PP substrate, making electrical contact with the selected device electrodes. The entire setup is enclosed in an electrically grounded Faraday box and maintained under a vacuum of approximately 5 × 10^–6^ mbar.

In panel b, two microheater actuators are visible, with four contact channels in the middle defining positions where distinct transition metal dichalcogenides (TMDCs) can be placed. The microheaters locally heat the polymer substrate, and as a result of its thermal expansion, an in-plane biaxial strain can be induced in the transferred 2D samples.^32,33^ Further details on the fabrication and performance of the microheater actuators are provided in ref 33.

In panel c, the calibration curve (see the Supporting Information for details on the calibration) demonstrates the dependence of the local temperature (and corresponding strain) on the applied microheater current squared (mA^2^) for two channel positions, marked in orange (spot 1) and blue (spot 2) in panel b. The data were obtained by monitoring the exciton shift in MoS_2_ in these two spots upon microheater biasing and comparing it with the response obtained upon heating the whole device with a macroscopic heater. The temperature changes at both spots remain approximately the same due to the device configuration used, where two microheater actuators are positioned on opposite sides.

The device enables fast response, up to 8 Hz,^33^ and control on local temperature variations of up to 30 °C, inducing reversible biaxial strain of approximately 0.4%, which relates to spectral red shifts of up to 20 nm. To ensure device stability, multiple devices were tested to determine a safe operating limit of 60 mA^2^ for the microheater bias, beyond which irreversible damage (melting of the surrounding PP) may occur, as illustrated in the optical image of the inset in panel c. Within this range, the microheater circuit maintains resistivity variations below 3%, enabling controlled and reproducible strain modulation by adjusting only the circuit current, as the Joule heating power is proportional to the square of the applied current (Supporting Information Figure S2).

Figure 2 details the optoelectronic performance of the strain-tunable photodetector. Panel a provides a close-up microscopy image of a typical device, showing 2D semiconductor flakes bridging the source–drain electrodes close to the nearby microheater. This configuration ensures efficient strain transfer and supports on-chip integration. We initially transferred four distinct TMDCs flakes onto the four channels, aiming to use distinct materials with their A exciton features spanning a broad range of the spectrum. However, the MoS_2_ and MoSe_2_ flakes (channels 1 and 4) exhibited insufficient signal-to-noise ratio and lacked temporal stability after the transfer process (see Supporting Information Figure S5). As a result, they were excluded from this study. In this particular device, only the WS_2_ and WSe_2_ samples (channels 2 and 3) remained functional after fabrication. Therefore, the microspectrometer footprint can be estimated as 40 × 20 μm^2^, corresponding to the active area of the WS_2_ and WSe_2_ samples.

**Figure 2:**
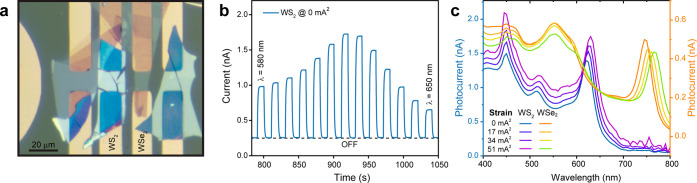
(a) Close-up optical microscopy image of a fabricated photodetector, illustrating the device architecture including the 2D semiconductors bridging the source–drain electrodes and the adjacent microheater. (b) Current vs time measured on the WS_2_ channel 2 (unbiased heaters) while the device is illuminated with different wavelengths to extract its spectral response. For each wavelength the illumination is switched ON and OFF, allowing one to directly measure the photocurrent, and then the wavelength is varied for the next ON/OFF cycle. (c) Photocurrent spectra responses for both WS_2_ (blue) and WSe_2_ (orange) recorded under four different microheater bias conditions, showing the spectral shift of the excitonic peak as a function of induced strain in the 2D material.

The dynamic response of the photodetector was evaluated by recording the current at a fixed bias voltage between drain and source electrodes as a function of time, while the device is illuminated with different wavelengths. The current readings were made one by one, selectively applying bias only to the electrodes associated with the channel being actively measured while keeping all other channels electrically floating. As depicted in Figure 2b, the time-resolved current obtained during incident monochromatic light [full width at half-maximum (fwhm) ∼ 8 nm] ON/OFF cycles reveals a fast and reproducible switching behavior, illustrating the stability and high signal-to-noise performance of the device. The current with light OFF is subtracted from the one with light ON to calculate the photocurrent for each incident light wavelength from 400 to 800 nm, with steps of 5 nm. The device’s spectral response for each channel and strain induced is then recorded.

In Figure 2c, the photocurrent spectra responses measured at different microheater bias levels are presented for two channels: WS_2_ and WSe_2_. The data illustrate a clear spectral shift: with increasing strain, the excitonic peak in the photocurrent response systematically shifts, confirming that thermal actuation effectively modulates the band structure of the 2D semiconductors. This tunability of the spectral response is a crucial feature for the adaptive functionality of the photodetectors.

Building on the strain-tunable photodetector platform, we further explored its application in reconstructive spectral sensing. Rather than employing the conventional matrix inversion techniques used in many gate-tunable van der Waals heterostructure spectrometers, our approach exploits the strain-induced spectral shifts to encode spectral information. To achieve spectral reconstruction, we developed a neural network framework trained on an extensive synthetic data set. Figure 3 schematically illustrates the process of device calibration, AI model training, and performance evaluation. Each device channel is based on a distinct 2D material. For each channel, the spectral response is experimentally recorded under different strain conditions, as induced by specific microheater biases, thereby establishing the device calibration, as demonstrated in Figure 2c for two channels and four strain levels.

**Figure 3:**
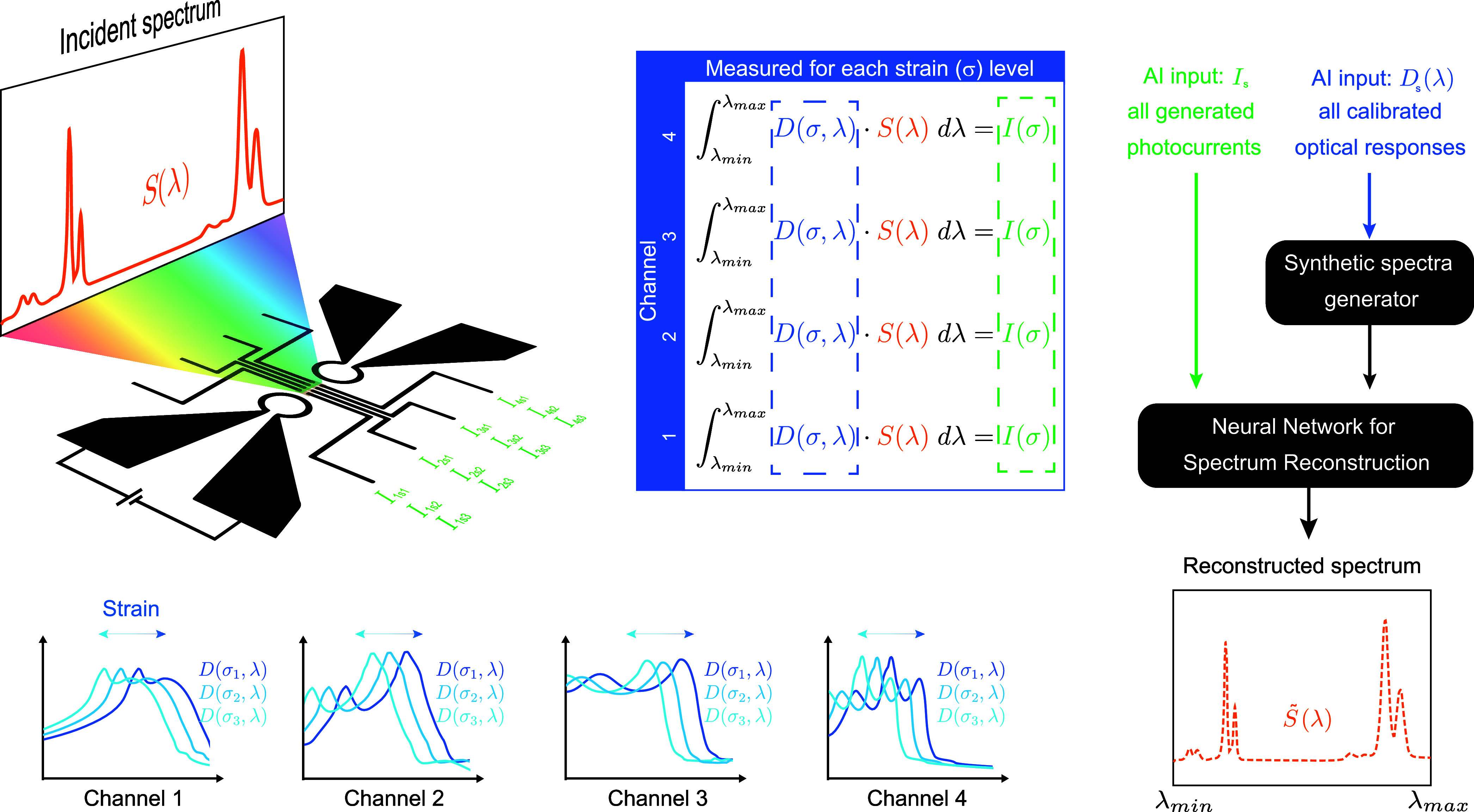
Schematic reconstruction process. Spectral responsivity *D* is experimentally characterized as a function of both wavelength and strain state (see Figure 2c). Using this information, it is possible to predict the resulting photocurrents when the device is exposed to an arbitrary spectrum. We exploit this to build a training database consisting of pairs of synthetic spectra and their predicted photocurrent response, which we use to train the reconstruction neural network to reconstruct the incident light spectrum.

Since acquiring a large experimental data set of different incident test spectra for all channels across the full range of strain conditions was impractical, we generated synthetic spectra and corresponding photocurrent responses for AI model training. For each synthetic spectrum, a matching set of photocurrent values was computed by integrating the spectrum with the calibrated spectral response of all channels at different strain levels, effectively simulating the expected device outputs for a broad range of input spectra. A total of 30000 synthetic photocurrent sets, each paired with its corresponding synthetic spectrum, was used to train the neural network model, together with an additional 10000 for validation and a further 10000 for testing. Details on the generation of synthetic spectra and the neural network architecture used can be found in Materials and Methods and will be published in more detail elsewhere.

In practice, an unknown incident spectrum generates a set of photocurrent values across *n* channels for each strain level *s*. This results in a data set of *n* × *s* photocurrent values, which serves as input for the AI model to reconstruct the original spectrum. A key parameter in this process is spectral discretization, which defines how the spectrum is divided into discrete intervals for accurate reconstruction. This approach allows for tuning the reconstruction resolution as a function of the model’s training time and complexity.

Figure 4 illustrates the proof-of-concept for the proposed reconstructive microspectrometer. The device, also shown in Figure 2, operates with only two channels, WS_2_ and WSe_2_. The spectral response calibration was performed for four strain levels, induced by applying 0 mA^2^ (∼0% strain), 17 mA^2^ (∼0.12% strain), 34 mA^2^ (∼0.24% strain), and 51 mA^2^ (∼0.36% strain) to the microheater, resulting in a spectral shift of 16 nm (20 nm) for WS_2_ (WSe_2_) within their respective A exciton spectral ranges.

**Figure 4:**
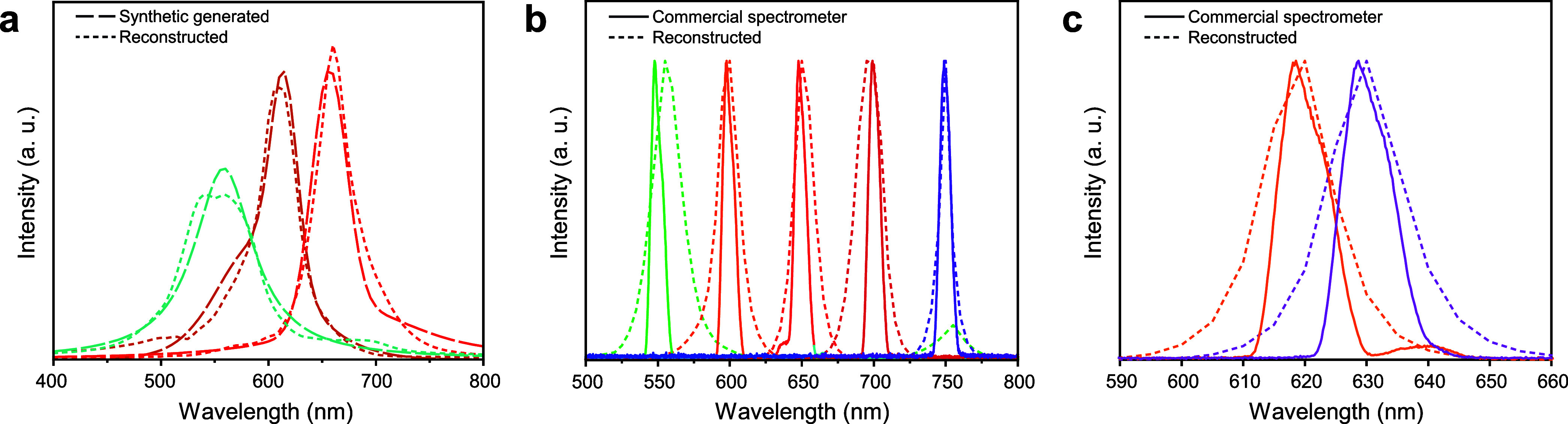
Testing the device with two channels (WS_2_ and WSe_2_) and four strain levels. (a) Synthetic generated spectra (long-dashed curves) and their reconstructed spectra (short-dashed curves). (b and c) Reconstructed spectra (short-dashed curves) made by the neural network applied to real photocurrent measurements, compared to the incident light spectra acquired with a commercial spectrometer (solid curves).

After training, the neural network model successfully reconstructed synthetic spectra with high accuracy, as shown in Figure 4a. For real incident spectra (monochromatic peaks with ∼8 nm fwhm centered at 550, 600, 650, 700, and 750 nm—the model was able to reconstruct single peaks that are closely spectrally aligned with the actual ones. As expected, spectra in the regions of the A excitons of the employed 2D materials (∼630 and ∼750 nm, respectively) were reconstructed more accurately than those outside that spectral region. It is noteworthy that the peak at 550 nm (green) has a somewhat shifted reconstruction and also shows an artifact secondary peak at 750 nm. The quality of reconstruction in different spectral regions depends on the information content available in the device’s responsivity, which, as can be appreciated in Figure 2c, is not homogeneous throughout the whole spectral range. Additionally, in future work we plan to explore training strategies for the neural network that incorporate a penalty for the output reconstructed spectra. This could help suppress artifacts and nonphysical features, leading to cleaner and more reliable spectral outputs.

Although only two channels and four strain levels were used, the results convincingly demonstrate that strain-induced tuning can serve as an effective mechanism for encoding spectral information in miniaturized spectrometers. In another test-of-concept, shown in Supporting Information Figure S7, only one material (WS_2_) in one channel under six strain levels could be used for spectral reconstruction in the A exciton range vicinity.

While the current implementation achieves spectral reconstruction over a limited wavelength range—corresponding to the extent of the excitonic shift, this proof-of-concept lays the foundation for further advancements. A promising avenue for future work is the integration of additional 2D semiconductor channels with complementary bandgaps. For instance, employing multiple TMDCs across different electrodes could effectively broaden the spectral encoding range and improve the reconstruction resolution and accuracy. Such a multichannel approach would allow each material’s intrinsic spectral response to cover distinct segments of the spectrum, thereby enabling a more comprehensive and higher-resolution spectral analysis. Additionally, using a monochromatic light source with narrower fwhm peaks than those employed in this study, along with a finer energy step calibration, could further enhance the resolution of the proposed miniaturized spectrometer.

In summary, we have demonstrated a novel strain-engineered photodetector platform that leverages the unique mechanical and electronic properties of 2D semiconductors for adaptive spectral sensing. Integrated microheaters enable precise thermal actuation, inducing strain that modulates the band structure and shifts the spectral response of the devices. By harnessing these strain-induced shifts, we developed a reconstructive microspectrometer, with a footprint of only 40 × 20 μm^2^, using a neural network trained on data derived from combining synthetic spectra and the experimental photocurrent spectral response of each device’s channel and strain induced level. Although the current implementation reconstructs spectra over a limited range, our results provide a compelling proof-of-concept for miniaturized adaptive spectroscopic systems. Future work will focus on further enhancing the spectral range and resolution through an improved device architecture and integration of multiple 2D material channels, paving the way for next-generation compact spectral sensors.

## Materials and Methods

### Materials

Four different two-dimensional (2D) semiconducting materials were employed in this study: molybdenum disulfide (MoS_2_) sourced from a natural mineral (Molly Hill Mine, Canada) and tungsten disulfide (WS_2_), tungsten diselenide (WSe_2_), and molybdenum diselenide (MoSe_2_) obtained from HQ Graphene. The substrate used for device fabrication was 500 μm thick PP (PP30-FM-000340, Goodfellow Cambridge Ltd.).

### Optoelectronic Characterization

The optoelectronic performance of the devices was characterized in a custom-built vacuum electrically grounded chamber equipped with a home-built probe card. The incident light shined on the sample through a borosilicate window in the chamber. All measurements were performed under vacuum conditions of approximately 5 × 10^–6^ mbar, after maintaining the system under vacuum for at least 24 h. An initial calibration measurement applying all strain levels, used to allow the device to stabilize, is performed but not considered in the final analysis. Subsequent calibration and testing steps are then carried out on the settled device. The incident light power density ranged from 11 to 38 μW/cm^2^. To ensure consistency across the spectral range, the measured photocurrents for the device’s calibration were normalized to a constant incident power, considering the linear photocurrent response with light intensity up to hundreds of mW/cm^2^ for this device architecture.^31^

A wavelength-adjustable light source (Bentham TLS120Xe) was used to calibrate the spectral response of the photodetectors over the wavelength range of interest. This light source produces peaks with an approximate fwhm of ∼8 nm, and its intensity varies with the wavelength. Therefore, the measured photocurrent response as a function of wavelength was corrected to account for a constant-intensity light source.

Photocurrent measurements were carried out by using a Keithley 2450 source measure unit, which provided both voltage biasing and current readout functions during the characterization experiments. Different drain–source voltages (*V*_SD_) were applied depending on the specific sample, with values chosen based on current–voltage (*I*–*V*) curve analysis to optimize both signal-to-noise and signal-to-background ratios. For the device shown in Figures 2 and 4, a *V*_DS_ of 0.1 V was applied to both the WS_2_ and WSe_2_ sample channels.

### AI Model Training

Randomly generated synthetic spectra were obtained as follows. We considered spectra with a random number of peaks ranging between 1 and 3. For a given spectrum, once the number of peaks had been selected, peak positions were chosen from a uniform distribution spanning the spectral range (400–800 nm, in this case), and likewise, full widths at half-maximum were also chosen for each peak, ranging between 5 and 50 nm. The shape of each peak was equally chosen randomly from three alternative possibilities, namely, a Lorentzian, a Gaussian, or a Voight shape. Lastly, once a random spectrum had been generated in this way, we discarded it if the intensity at either limit of the spectral range was greater than 5% of the maximum intensity within the range. If a synthetic spectrum was deemed acceptable, the photocurrents it would produce were calculated by multiplying it by each of the measured photocurrent response curves of WS2 and WSe_2_ (see Figure 2c), followed by integration over the spectral range, as indicated in the upper central panel of Figure 3.

For the neural network we adopted a multilayer perceptron design, with an input layer of size equal to the number of detector channels multiplied by the number of strain levels (8, in this case). The input layer was followed by a number of deep layers with a variable number of neurons per layer, interspersed by a nonlinear activation (we used the ELU activation function). In actual practice we used four deep layers with 41 neurons per layer; although we did not perform a systematic optimization of these hyperparameters, we did check that varying them around these default values resulted in no appreciable change. The number of neurons in the final output layer determines the spectral resolution; in this work, we imposed a spectral resolution of 5 nm, which required 81 neurons in the output layer. So as to enforce the normalization of the resulting spectrum, a SoftMax layer was used. The model was implemented using Pytorch,^34^ and relied on the scikit-fda Python package.^35^

### Formvar Polymer Encapsulation

To enhance device stability and thermal-induced strain actuation, a recently developed formvar encapsulation technique was applied following device fabrication and samples transfer.^31^ This encapsulation step plays a critical role in preserving the mechanical integrity of the 2D materials during thermal cycling. Moreover, it increases the strain gauge factor by approximately 2-fold, resulting in significantly larger excitonic peak energy shifts under the same temperature variation applied to the PP substrate. It has also been reported that formvar encapsulation increases the maximum achievable strain from approximately 1.4 to 2.3%, and devices fabricated on PP substrates demonstrate enhanced optoelectronic performance when encapsulated with formvar, including higher and faster photocurrent response, as well as improved device longevity. For the encapsulation step, 50 μL of formvar solution (*∼*1% (w/w) polyvinyl formal in 1,2-dichloroethane, formvar solution from Merck Co.) was spin-coated (3000 rpm and 40 s) using an Ossila spin coater onto the device surface and left to dry for 24 h under ambient conditions (22 °C and 45% RH).

## Supplementary Material



## Notes

Device fabrication, 2D flake exfoliation and transfer, and formvar polymer encapsulation method; analysis of number of flakes layers; complete calibration data; test of device with only one channel (PDF)

## ∥

Instituto de Energia Solar, Universidad Politécnica de Madrid, 28040 Madrid, Spain

## ⊥

Department of Materials Science and Engineering, University of California, Berkeley, CA, USA.
