# Supplementary material for: Strain-Engineered Adaptive 2D Photodetectors: A New Approach to Miniaturized Reconstructive Spectrometry
Source: Nano Lett. 2025 Jul 12;25(29):11333–9. doi: 10.1021/acs.nanolett.5c02470 (PMC12291583; doi:10.1021/acs.nanolett.5c02470)
Supplement: Supplementary file 1 [file nl5c02470_si_001.pdf]

# Supporting Information

# Strain-engineered adaptive 2D photodetectors: a new approach to miniaturized reconstructive spectrometry

*Thiago L. Vasconcelos*[1,2,*], *Simeon N. Vladimirov*[1], *Thomas Pucher*[1], *Sergio Puebla*[1, †], *Carmen Munuera*[1], *Eduardo R. Hernández*[3,*] *and Andres Castellanos-Gomez*[1,*]

[1]2D Foundry research group. Instituto de Ciencia de Materiales de Madrid (ICMM-CSIC), Madrid, E-28049, Spain.

[2]Materials Metrology Division, Instituto Nacional de Metrologia Qualidade e Tecnologia (INMETRO), Duque de Caxias-RJ, 25250-020, Brazil.

[3]Computational Materials Laboratory, Instituto de Ciencia de Materiales de Madrid Madrid (ICMM-CSIC), Madrid, E-28049, Spain.

* Corresponding Authors: tlvasconcelos@inmetro.gov.br, eduardo.hernandez@csic.es, andres.castellanos@csic.es

**Device fabrication**

Device fabrication was performed using maskless photolithography (SmartPrint by Microlight3D) with a bilayer photoresist system consisting of LOR-2A (Kayaku Advanced Materials Inc.) and AZ 1505 (MicroChemicals GmbH) positive resists. To ensure a uniform and planar surface for subsequent microfabrication steps, 20×20 $mm^2$ PP substrates were first planarized by heating them to 145 °C while pressing them between two silicon wafers under a 500 g weight.

After cooling, the LOR-2A layer was spin-coated at 3000 rpm for 40 s and baked at 130 °C for 5 minutes. Once cooled, AZ 1505 was spin-coated on top at 2500 rpm for 20 s and baked at 100 °C for 60 s. Following UV exposure of the device pattern, the sample was developed in AZ 726 MIF developer for 19 s. A post-exposure bake was then performed at 100 °C for 5 minutes, followed by a second development step in AZ 726 MIF for 15 s to form an undercut profile by selectively removing the LOR beneath the hardened resist. Finally, the sample was rinsed in deionized water and dried under a nitrogen stream.

Metal electrodes were deposited using electron-beam evaporation. First, a 5 nm titanium adhesion layer was deposited at a rate of 0.2 Å/s, followed by a 45 nm gold layer deposited at 0.5 Å/s. The lift-off process was performed to remove the photoresist and LOR layers, revealing the patterned metal electrodes on the PP substrate. This was achieved by sequentially immersing the sample in acetone and then in a DMSO solution and cleaned with DI water, ensuring complete removal of the resist and clean definition of the metal structures.

## Exfoliation and transfer of 2D semiconductors flakes

The 2D crystals were mechanically exfoliated using Nitto SPV224 tape (Nitto Denko). Following exfoliation, the flakes were initially transferred onto Gel Film WF 4×6.0 mil (Gelpak) to facilitate optical identification. Suitable flakes were selected using transmission mode optical microscopy (Motic BA310 MET-T) and differential microreflectance spectroscopy.[1,2] A dry deterministic transfer method was then employed to place the selected flakes onto the final device substrate, bridging pre-patterned source and drain electrodes as described in Ref.[3] In this specific work, deterministic transfers were performed individually for each of the four different materials. During the transfer of each new flake, a relatively high release temperature of 60 °C was used to ensure that the PDMS stamp did not pick up or disturb previously transferred flakes on the substrate. In the device shown in Figure 2, a seven-layer $WS_2$ flake and a three-layer $WSe_2$ flake were transferred to channels 2 and 3, respectively, along with four-layer $MoS_2$ flake and six-layer $MoSe_2$ flake in channels 1 and 4, respectively (see Supporting Information Figure S1). Unfortunately, the photocurrent stability in these last two channels was insufficient for reliable use, possibly due to contact issues between the transferred materials and the gold electrodes, or damage to the channels during transfer. Therefore, they were excluded from this study. One possible strategy to address this in future devices is to perform AFM ironing to improve the contact between the flakes and the electrodes prior to the Formvar encapsulation process, as reported in Ref.[4]

## Layer number analysis of transferred TMDCs samples

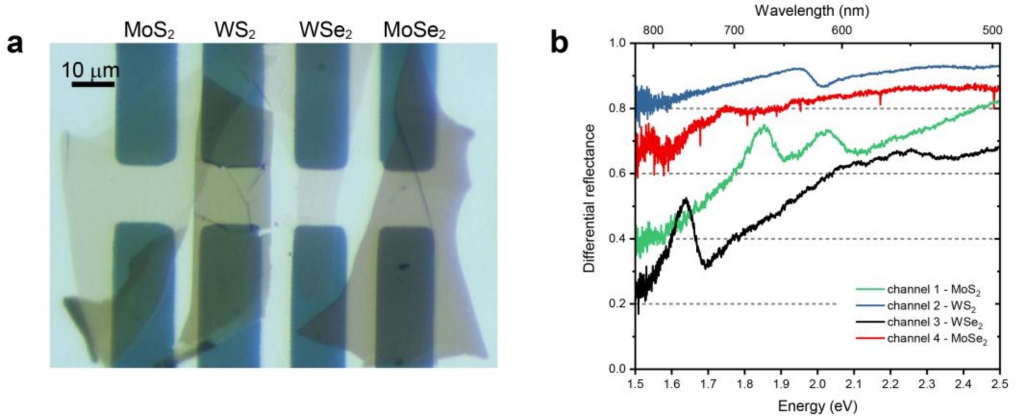


**Figure S1:** Panel (a), transmitted optical microcopy image of the device shown in Figure 2(a), highlighting sample thicknesses. Panel (b), Differential microreflectance spectra obtained from the four transferred samples, indicating (based on Ref.[2]): four-layer $MoS_2$ at channel 1, seven-layer $WS_2$ in channel 2, three-layer $WSe_2$ in channel 3, and six-layer $MoSe_2$ in channel 4.

## Safe current limits of microheater actuator

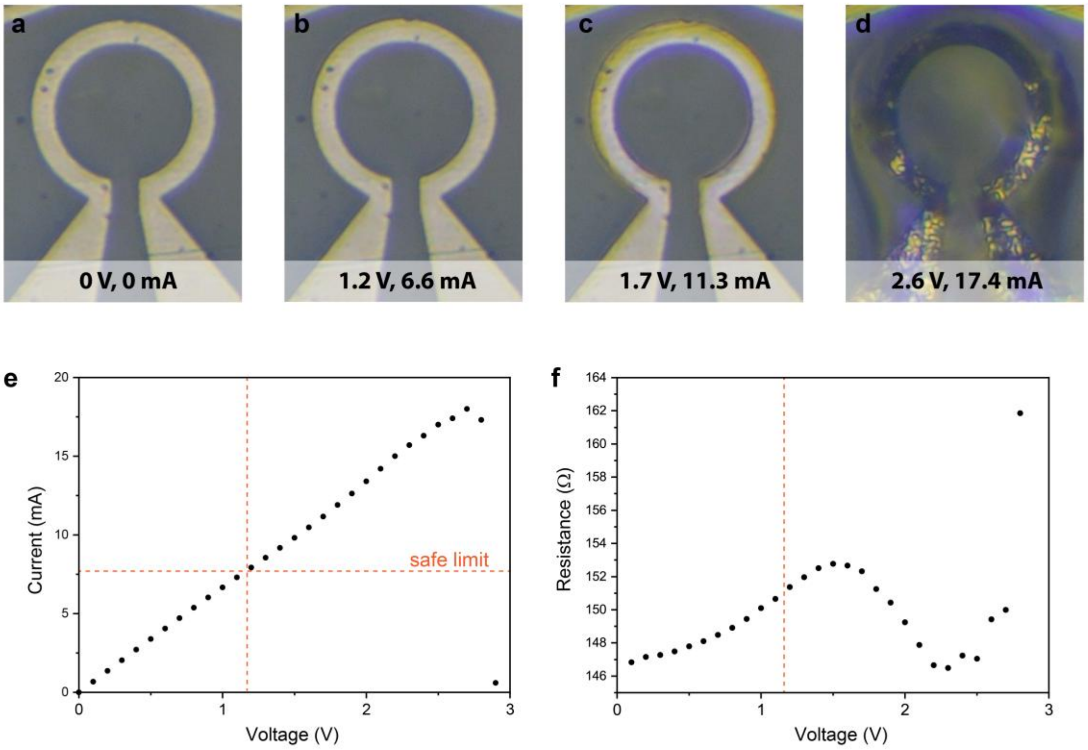


**Figure S2:** Study of the safe operating current range for the dual microheater actuator. Optical images were captured at each 0.1 V increment of applied voltage. (a–d) Optical images of the device reveal a visible color change at around 1.7 V (11.3 mA) and signs of permanent damage, likely due to PP substrate melting, at around 2.6 V (17.4 mA). (e) The current–voltage (I–V) curve shows linear behavior up to approximately 2.7 V. (f) A drop in resistance is observed around 1.5 V (~10 mA), indicating the onset of device degradation. Based on the analysis of multiple devices, a safe operating current limit was established at 7.75 mA (equivalent to 60 $mA^2$). Within this range, the circuit resistance varied by no more than 3%, and no signs of damage were observed in either the electrical characteristics or the optical images.

**Calibration of the device microheater actuator**

Microheaters were integrated adjacent to the photodetector structures. Calibration of the microheaters was performed following the method described in Ref.[5]. Briefly, the exciton shift of a large $MoS_2$ monolayer flake, positioned between the two microheater actuators, was monitored by differential microreflectance spectroscopy as a function of the applied microheater current squared ($mA^2$) (see Supporting Information Figures S3 and S4). These measurements were then compared to those obtained by uniformly heating the entire sample using a macroscopic heater. This comparison enabled accurate determination of the temperature reached at the $MoS_2$ flake during microheater actuation

For the strain measurements shown in the plot of Figure 1c, the correlation between biaxial strain in a monolayer $MoS_2$ flake on a PP substrate and the applied temperature variation, as previously determined in Ref. [6] was used. Specifically, the strain was calculated using the relation: $\Delta\epsilon = -0.357 + 0.0130 \times \Delta T$, where $\epsilon$ is the biaxial strain (%) and T is the temperature in °C. In addition, a uniaxial strain experiment was performed on the same sample to evaluate its relationship with the applied microheater current (Supporting Information Figure S4). This results reveal that uniaxial strain values could reach up to twice those calculated for the biaxial strain case, the one that better describe the thermal induced strain and as also observed in Ref.[7] All these experiments were conducted at two specific spots corresponding to channels 3 and 4, as indicated in Figure 1b and Figure S3.

To evaluate the homogeneity of the strain induced across different channels regions under varying microheater actuation conditions, we complemented the previous results obtained via differential microreflectance spectroscopy with photoluminescence (PL) mapping performed at different applied microheater currents (see Supporting Information Figure S6). These measurements revealed a consistent excitonic shift of approximately 65 meV under a microheater actuation of 50 $mA^2$, with only minor variations up to 5 meV depending on the specific position referent to each channel.

All differential microreflectance spectral fitting and PL hyperspectral map fit were performed using FabNS's PortoFlow Analysis Software.

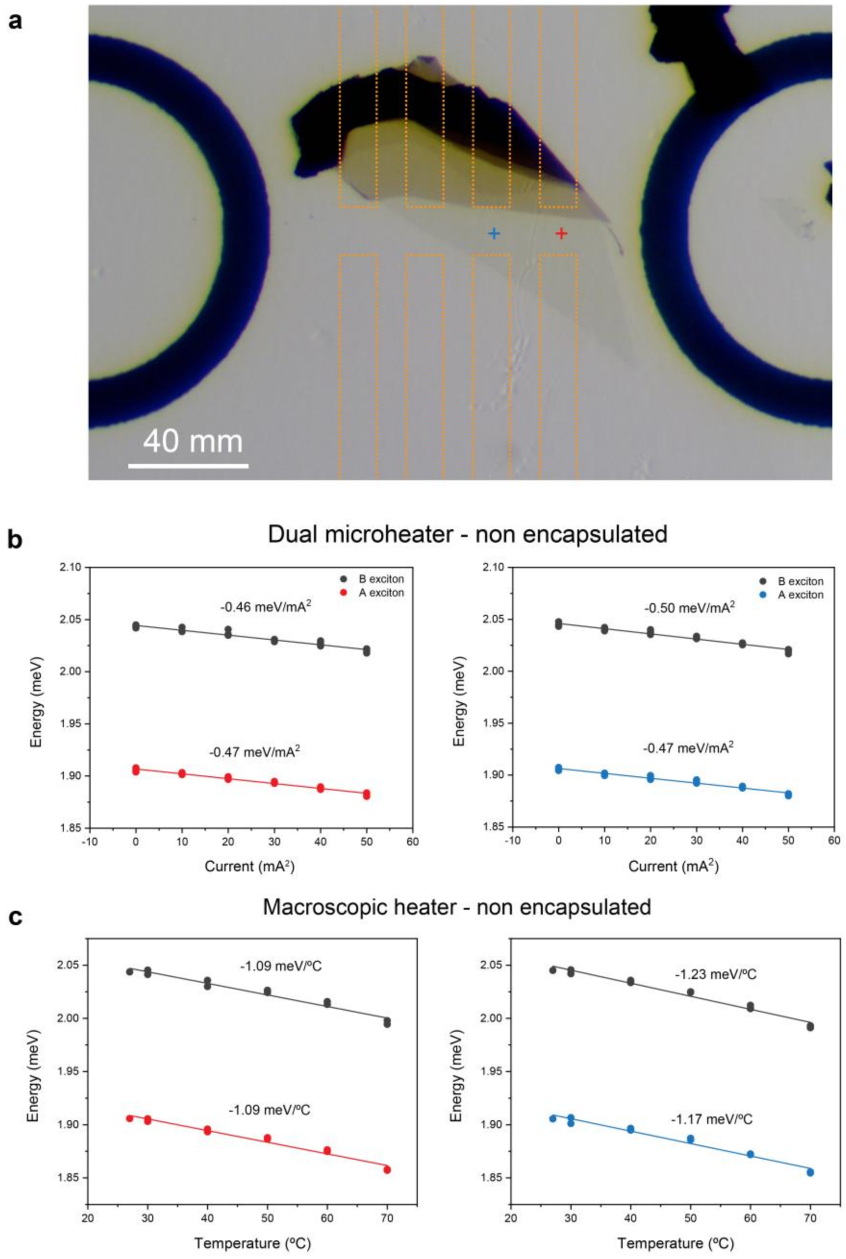


**Figure S3:** Differential microreflectance measurements of a device with only the dual microheater actuator contacts on a PP substrate and a large monolayer $MoS_2$ sample in between them. The positions of the channel contacts are marked in orange dashed lines in panel (a). Reflectance was analyzed at two points corresponding to the active area of contact channels 3 [blue marker in (a)] and 4 [red marker in (a)]. The graphs display the peak energy of the A (colored in red/blue) and B (black) excitons under strain induced by: the dual microheater actuator and macroscopic heater (entire device heated), before Formvar encapsulation.

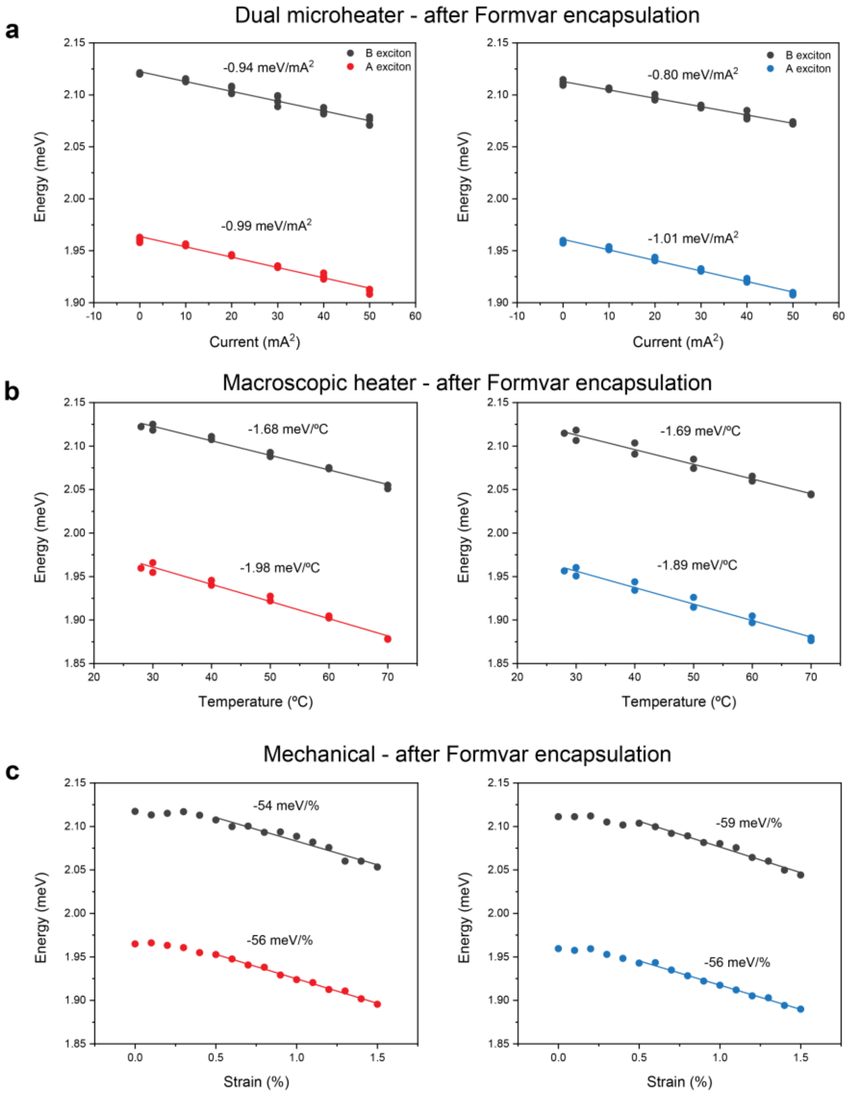


**Figure S4:** Differential microreflectance measurements taken at two locations corresponding to the active areas of contact channels 3 (blue marker) and 4 (red marker), as indicated in Figure S3(a). The plots show the energy position of the A exciton (colored red and blue) and B exciton (black) under three different strain-inducing conditions: (a) dual microheater actuation, (b) uniform thermal macroscopic heating of the entire device, and (c) mechanically applied uniaxial strain. All measurements were performed after Formvar polymer encapsulation.

**Spectral Response of Channels 1 and 4 from the device shown in Figure 2**

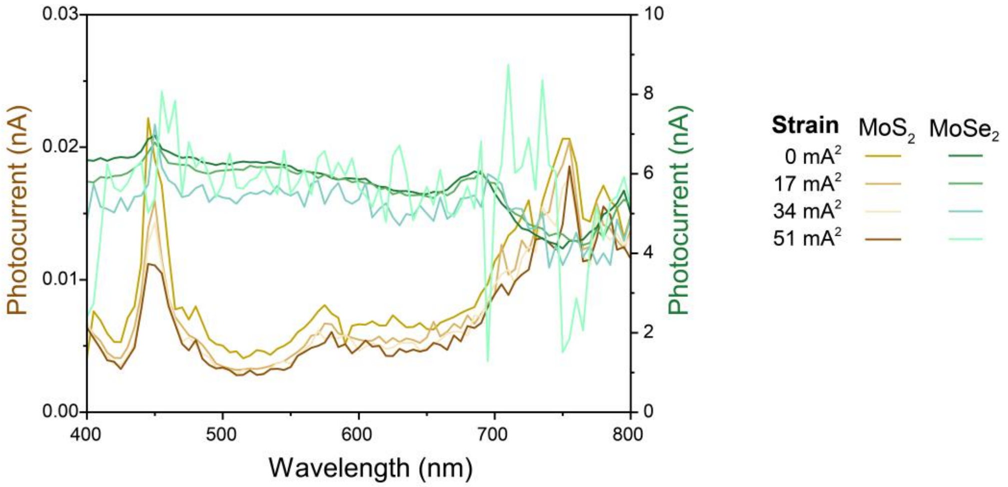


**Figure S5:** Photocurrent spectra response for the channels 1 ($MoS_2$ in brown) and 4 ($MoSe_2$ in green) of the device of Figure 3 recorded under four different microheater bias conditions. Both channels exhibited poor performance, with $MoS_2$ showing very low photocurrent and both materials displaying insufficient signal-to-noise ratios. Due to these limitations, they were excluded from further analysis in this study.

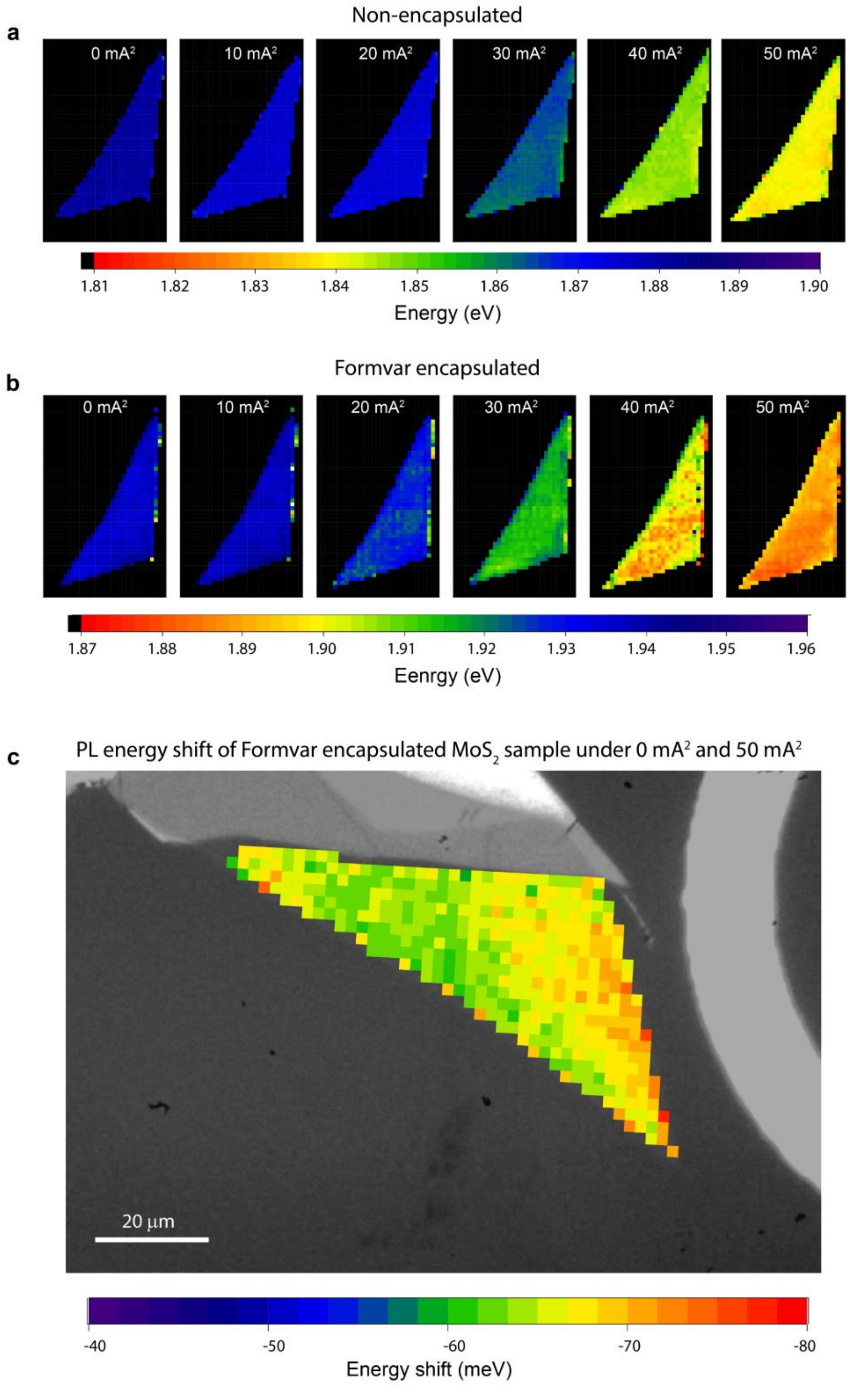


**Figure S6:** Photoluminescence (PL) hyperspectral imaging results of a device containing only the dual microheater actuator contacts on a PP substrate, with a large monolayer $MoS_2$ flake positioned across the channels gap region. (a, b) PL energy maps showing the spatial distribution of the A exciton peak position under strain induced by the dual microheater actuator at 0, 10, 20,

30, 40, and 50 mA$^2$, recorded before (a) and after (b) Formvar polymer encapsulation. The color scale represents the energy position of the A exciton peak. (c) Difference map obtained by aligning and subtracting the PL energy maps at 0 mA$^2$ and 50 mA$^2$ after Formvar encapsulation, highlighting the local A exciton energy shift across the sample due to the induced strain.

## Test of a device with only one channel under six strain levels

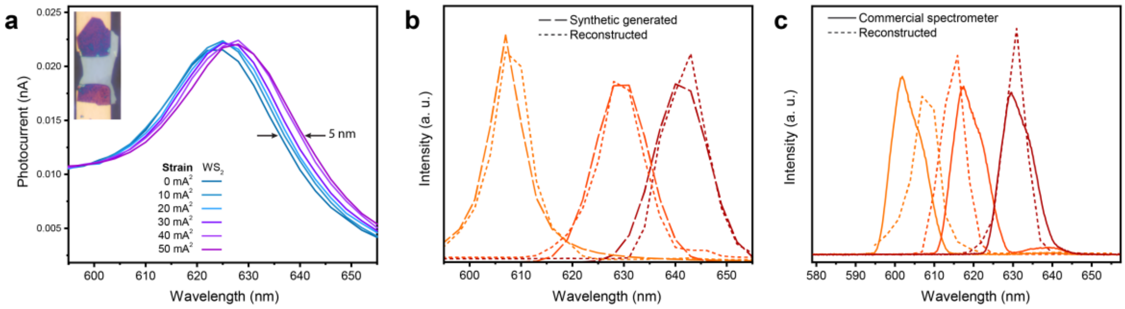


**Figure S7:** (a) Photocurrent spectral response of the $WS_2$ channel under six different microheater bias conditions, showing a clear spectral shift of the excitonic peak as a function of strain induced by the dual microheater actuator (from 0 mA$^2$ to 50 mA$^2$ in 10 mA$^2$ steps). Inset: close-up optical microscopy image of the fabricated photodetector made with around seven-layer $WS_2$ sample. (b) Device test using a single $WS_2$ channel and six strain levels: synthetic spectra (long-dashed curves) and corresponding reconstructed spectra (short-dashed curves) generated by the neural network. (c) Reconstructed spectra (short-dashed curves) obtained from real photocurrent measurements using the trained neural network, compared with the incident light spectra recorded by a commercial spectrometer (solid curves).

## REFERENCES


(1) Taghavi, N. S.; Gant, P.; Huang, P.; Niehues, I.; Schmidt, R.; Vasconcellos, S. M. De; Bratschitsch, R.; García-hernández, M.; Frisenda, R.; Castellanos-Gomez, A. Thickness Determination of MoS 2, MoSe 2, WS 2 and WSe 2 on Transparent Stamps Used for Deterministic Transfer of 2D Materials. *Nano Res.* **2019**, *12* (7), 1691–1695. https://doi.org/https://doi.org/10.1007/s12274-019-2424-6.

(2) Niu, Y.; Gonzalez-Abad, S.; Frisenda, R.; Marauhn, P.; Drüppel, M.; Gant, P.; Schmidt, R.; Taghavi, N. S.; Barcons, D.; Molina-Mendoza, A. J.; de Vasconcellos, S. M.; Bratschitsch, R.; De Lara, D. P.; Rohlfing, M.; Castellanos-Gomez, A. Thickness-Dependent Differential Reflectance Spectra of Monolayer and Few-Layer MoS2, MoSe2, WS2 and WSe2. *Nanomaterials* **2018**, *8* (9). https://doi.org/10.3390/nano8090725.

(3) Castellanos-Gomez, A.; Buscema, M.; Molenaar, R.; Singh, V.; Janssen, L.; Van Der Zant, H. S. J.; Steele, G. A. Deterministic Transfer of Two-Dimensional Materials by All-Dry Viscoelastic Stamping. *2D Mater.* **2014**, *1* (1), 11002–11010. https://doi.org/10.1088/2053-1583/1/1/011002.

(4) Palai, S. K.; Dyksik, M.; Sokolowski, N.; Ciorga, M.; Sánchez Viso, E.; Xie, Y.; Schubert, A.; Taniguchi, T.; Watanabe, K.; Maude, D. K.; Surrente, A.; Baranowski, M.; Castellanos-Gomez, A.; Munuera, C.; Plochocka, P. Approaching the Intrinsic Properties of Moiré Structures Using Atomic Force Microscopy Ironing. *Nano Lett.* **2023**, *23* (11), 4749–4755. https://doi.org/10.1021/acs.nanolett.2c04765.

(5) Ryu, Y. K.; Carrascoso, F.; López-Nebreda, R.; Agraït, N.; Frisenda, R.; Castellanos-Gomez, A. Microheater Actuators as a Versatile Platform for Strain Engineering in 2D Materials. *Nano Lett.* **2020**, *20* (7), 5339–5345. https://doi.org/10.1021/acs.nanolett.0c01706.

(6) Carrascoso, F.; Lin, D. Y.; Frisenda, R.; Castellanos-Gomez, A. Biaxial Strain Tuning of Interlayer Excitons in Bilayer MoS2. *JPhys Mater.* **2020**, *3* (1). https://doi.org/10.1088/2515-7639/ab4432.

(7) Carrascoso, F.; Frisenda, R.; Castellanos-Gomez, A. Biaxial versus Uniaxial Strain Tuning of Single-Layer MoS2. *Nano Mater. Sci.* **2022**, *4* (1), 44–51. https://doi.org/10.1016/j.nanoms.2021.03.001.
